# Depicting Medullary Thyroid Cancer Recurrence: The Past and the Future of Nuclear Medicine Imaging

**DOI:** 10.5812/ijem.8156

**Published:** 2013-10-01

**Authors:** Evangelia Skoura

**Affiliations:** 1 Nuclear Medicine Department, Evangelismos Hospital, Athens, Greece

**Keywords:** Medullary Thyroid Cancer-MTC, Calcitonin, Pentetreotide, 3-Iodobenzylguanidine, Positron-Emission Tomography, Fluorodeoxyglucose F18

## Abstract

**Context:**

Inherited and sporadic medullary thyroid cancer (MTC) is an uncommon and medically challenging malignancy. Even if the extent of initial surgery is deemed adequate, the recurrence rate remains high, up to 50% in most series. Measurement of serum calcitonin is important in the follow-up of patients with MTC, and reliably reflects the existence of the disease.

**Evidence Acquisition:**

There is no single sensitive diagnostic imaging method to reveal all MTC recurrences or metastases. Conventional morphologic imaging methods (U/S, CT, and MRI) and several methods of nuclear medicine have been used for this purpose with variable accuracy.

**Results:**

The main role of nuclear medicine imaging is the detection of residual or recurrent tumor in the postoperative follow-up. In this review we present the radiopharmaceuticals used in the diagnosis of MTC recurrence, and comparison among them.

**Conclusions:**

The most used radiopharmaceuticals labelled with γ emitters are: Metaiodobenzylguanidine (MIBG), labelled with ^131^I or ^123^I, ^111^In-pentetreotide (Octreoscan), 99mTc-pentavalent dimercaptosuccinic acid (^99m^Tc(V)-DMSA), and ^99m^Tc-EDDA/HYNIC-Tyr3-Octreotide ( Tektrotyd). The radiopharmaceuticals labelled with a positron-emitting radionuclide (β+), suitable for positron emission tomography (PET) imaging are: ^18^F-fluorodeoxyglucose (^18^F-FDG), ^18^F-fluorodihydroxyphenylalanine (18F-DOPA), and 68Ga-labelled somatostatin analogues (68Ga-DOTATATE or DOTATOC).

## 1. Introduction

Inherited and sporadic medullary thyroid cancer (MTC) is an uncommon and medically challenging malignancy which originates from the parafollicular calcitonin-secreting cells of the thyroid. MTC makes up 3%–10% of all thyroid cancers and 13.4% of all thyroid-related deaths (1, 2). Its low incidence is the limitation of both widespread clinical expertise and definitive randomized clinical trials (1). MTC may occur in sporadic (75% of cases) or hereditary (25% of cases) forms which include multiple endocrine neoplasia (MEN) types IIA and IIB, and isolated familial MTC (2). 

When no distant metastasis is present, the curative treatment for MTC is total thyroidectomy and lymph node dissection (1, 3). Nevertheless, the recurrence rate remains high, up to 50% in most series (1). Measurement of the serum calcitonin is important in the follow-up of patients with MTC, and reliably reflects the presence and volume of disease in most of them. Calcitonin levels should be measured 2–3 months postoperatively, as it has a half-life of about 30 h (3). When calcitonin is undetectable, a pentagastrin stimulation test may be performed to exclude any residual disease (1). If both the basal and the stimulated serum calcitonin are undetectable the patient is in complete biochemical remission and has about a 3% chance of biochemical recurrent disease during follow-up (4). It is reported that biochemical cure predicted a survival rate of 97.7% at 10 years (5). 

When MTC recurrence occurs, reoperation seems to be the only treatment strategy that, with good patient selection, can result in local disease control. Neither, conventional chemotherapy, nor external beam radiotherapy has a significant role in the treatment of these patients. Recent on-going trials with new classes of drugs, as tyrosine kinase inhibitors (e.g. Vandetanib and Cabozanitinib), have shown promising results (1). However, the most important prognostic factor in patients with recurrent MTC remains the early diagnosis, careful patient selection, and recognition of the recurrent lesions (3). 

Nowadays, there is no single sensitive diagnostic imaging method to reveal all MTC recurrences or metastases. Conventional morphologic imaging methods, neck ultrasound (U/S), cervical, thoracic, and abdominal computed tomography (CT), and thoracic and abdominal magnetic resonance imaging have been used for this purpose with variable accuracy (6). However, often MTC lesions are difficult to localize due to their small size and the reliable differentiation between scar tissue and recurrent tumor is frequently not possible (1, 6, 7). Ultrasonography (U/S) has shown a lymph node detection rate of 28%-78%, compared to 38%-70% and 44%-74% for CT and MRI, respectively (7). Generally, the combined use of various diagnostic procedures allows the identification of recurrent tumor in approximately 40% of patients (6). 

Several methods of nuclear medicine have been used for the detection of MTC recurrent lesions, especially when there are elevated levels of serum calcitonin and the conventional imaging has negative results for such purpose, a great number of radiopharmaceuticals, either for γ-camera or positron emission tomography (PET), have been proposed (8). The most difficult challenge was to find a technique with high sensitivity and specificity in detecting tumor remnant or relapse after surgery.

In this review we present the radiopharmaceuticals used in diagnosis of MTC recurrence (Table 1), although some of them, as radiolabelled monoclonal antibodies, can be considered either of historical or experimental value and the use of ^99m^Tc(V)-DMSA is going to be abandoned (8 , 9). 

**Table 1.** Radiopharmaceuticals of Choice for MTC Imaging

Radiopharmaceutical Labelled With a Radionuclide That Emits γ Radiation
**Radiopharmaceuticals most used**
**Metaiodobenzylguanidine (MIBG), labelled with 131I or 123I**
**111In-pentetreotide (Octreoscan)**
**99mTc-Depreotide (Neospect)**
**99mTc-EDDA/HYNIC-Tyr3-Octreotide (Tektrotyd)**
**Radiopharmaceuticals not usually used**
**99mTc(V)-dimercaptosuccinic acid (DMSA)**
**201Thalium (201Tl)**
**99mTc-sestamibi (MIBI)**
**Radiolabelled monoclonal anti-CEA antibodies (131I-anti-CEA antibody)**
**Cholecystokinin (CCK)-B/gastrin receptor imaging (e.g. 111In-DTPA-minigastrin, MG0)**
**Radiopharmaceutical labelled with a positron-emitting radionuclide ( β + )**
**18F-fluorodeoxyglucose (18F-FDG)**
**18F-fluorodihydroxyphenylalanine (18F-DOPA)**
**68Ga-labelled somatostatin analogues (68Ga-DOTATATE or DOTATOC).**

## 2. Radiopharmaceuticals

### 2.1. Single Photon Emission Tracers

#### 2.1.1. Technetium-99m Pentavalent Dimercaptosuccinic Acid, ^99m^Tc(V)-DMSA 

The main clinical role of ^99m^Tc(V)-DMSA was in patients with primary or recurrent MTC (10). According to published studies, its sensitivity ranged from 50% to 80% (11). The wide range of sensitivity could be explained by the use of different commercial kits, as a result of instability of the isomeric composition of their manufactured product (10). 

The ^99m^Tc(V)-DMSA imaging is valuable prior and after the surgical removal of MTC (12). In the preoperative setting the uptake of ^99m^Tc (V)-DMSA by the tumor is assessed. Postoperatively, it is used to evaluate the presence of residual disease. During follow-up period, ^99m^Tc(V)-DMSA could localize the site of recurrence when calcitonin level starts to increase (10).

The intensity of ^99m^Tc(V)-DMSA uptake in MTC lesions varies from minimal to high depending on the involved tissue, e.g. uptake in soft tissue sites appears more intense than in bone metastases (10). 

Nowadays the use of ^99m^Tc(V)-DMSA is going to be abandoned and in most countries it is not commercially available (9).

#### 2.1.2. Radioiodinated Metaiodobenzylguanidine (MIBG) 

Radioiodinated metaiodobenzylguanidine (MIBG), first used in 1980 for the imaging of the tumors of the adrenal medulla, is a radiopharmaceutical specific for tumors originating from the neural crest, including MTC (6). MIBG, resulting from the combination of the benzyl group of bretylium and the guanidine group of guanethidine, is a noradrenaline (norepinephrine) analogue. It is actively transported into noradrenaline granules of sympathetic nerve terminals by the noradrenaline transporter (type 1 uptake mechanism) (13). It exploits the type 1 uptake mechanism at the cell membrane, and is stored within intracellular storage vesicles MIBG can be labeled with either ^131^I- or ^123^I. Nowadays, both radiopharmaceuticals (^131^I- and ^123^I –MIBG) are available for diagnostic purposes but the use of ^123^I -MIBG is to be considered the agent of choice, as it has a more favorable dosimetry (159 KeV photon energy, T1/2=13.2h, fewer particulate emissions) and provides better image quality, better photon detection and greater sensitivity in tumor detection (8). Also, when ^123^I-MIBG is used, the higher photon flow allows high-quality single-photon emission computed tomography (SPECT) to be performed (13).

In 1984, the first positive case of ^131^I-MIBG uptake in a patient with MEN IIA was reported, where there was uptake in both the primary MTC and the coexistent pheochromocytoma (10). After this, the results of many series of MTC patients studied with MIBG have been published (11, 14). Although the results of the first studies were encouraging, the following results were controversial (15). A study showed a high rate of specificity, reaching 95% but sensitivity was much lower, only 30%, while other researchers have reported better detection results in familial rather than in sporadic MTC, and higher sensitivity in detection of residual or recurrent lesions in comparison with distant metastases (15). This wide range of sensitivity values might be related to the histological heterogeneity of MTC and the possible anaplastic transformation of the metastases. In the last years, new radiopharmaceuticals have been proposed for the imaging of MTC, and the use of MIBG is very limited.

#### 2.1.3. Somatostatin Receptor Scintigraphy (SRS)

Somatostatin is a regulatory peptide widely distributed in the human body. In the nervous system, somatostatin acts as a neurotransmitter, whereas its hormonal activities include the inhibition of the physiologic and tumorous release of growth hormone, insulin, glucagon, gastrin, serotonin, and calcitonin (16). Its other actions are an antiproliferative effect on tumors and also specific regulation of immune responses (16). Somatostatin action is mediated through membrane-bound receptors, of which five have been cloned (sst1-sst5), and they all belong to the family of G-protein-coupled receptors (17). Somatostatin is a peptide with two forms, containing 14 and 28 amino acids, respectively (17). Both bind to all subclasses of somatostatin receptors but are rapidly degraded in the blood by peptidases and have a short half life (Τ_1/2_=1-2 min) (17). Various synthetic somatostatin analogues have been made to increase resistance to peptidases and thereby allow systemic delivery by virtue of longer circulation times. These synthetic somatostatin analogues have varying affinity for the different types of somatostatin receptors (8). However, only sst2, sst5 and, to some extent, sst3 have a high affinity for the commercially available synthetic peptides, octreotide, lanreotide, and vapreotide (16). The most widely used is an 8-amino acid peptide, octreotide, with a half-life of T_1/2_= 2.83 days (17). This peptide has been radiolabelled as [^111^In] diethylenetriaminepentaacetic acid (DTPA)-octreotide (Octreoscan, Covidien, Petten, The Netherlands), and it has highest affinity for sstr2 and is suitable for imaging on a gamma camera (17).

Extremely variable results of Octreoscan in patients with MTC have been reported in the literature (6). The tumor detection rate ranges between 20% and 64% (8). According to a study, the sensitivity of Octreoscan is lower than this of conventional imaging (37%), but others support that the sensitivity is 50%-75% and higher than radiolabelled MIBG and similar or slightly superior to that ^99m^Tc (V)-DMSA (18-20). SPECT imaging may increase the lesion detection in the neck and liver. The results of in vitro studies have shown that the incidence of somatostatin receptors is higher in differentiated MTC lesions, as their density is directly related to tumor differentiation (21). These results have been confirmed by clinical studies showing that the sensitivity of Octreoscan is higher for detection of involved lymph nodes in patients with occult MTC, typically associated with less aggressive behaviour. On the opposite, this method is less sensitive in cases with distant metastases and progressive disease (22). 

Several attempts have been performed to label somatostatin analogues with ^99m^Tc, which is the most available radionuclide. So, ^99m^Tc-EDDA/HYNIC-Tyr^3^-Octreotide (Tektrotyd) scintigraphy seems to have significant sensitivity and specificity in MTC (23). It seems to be clinically useful in the follow-up of patients with MTC, with an overall sensitivity of 79.5%, and a specificity of 83.3% (24). 

Indication for radiolabelled somatostatin, besides the follow-up of patients with known disease, is also the selection of patients with inoperable tumors for peptide receptor radionuclide therapy with ^90^Y-DOTA^0^-Tyr^3^-octreotide (^90^Y-DOTATOC) or ^177^Lu-DOTA^0^-Tyr^3^-octreotate (^177^Lu-DOTA-TATE) (23). 

#### 2.1.4. ^201^ Thalium (^201^Tl)

^201^ Tl has been recognized as a tumor-imaging agent since 1976 (10). Accumulation of ^201^Tl in both primary and recurrent MTC lesions has been reported with sensitivity and specificity reaching up to 91% and 100%, respectively (25, 26). The main problem of the imaging with ^201^Tl is the nonspecific uptake in the liver and lungs which reduces its sensitivity in detecting metastases in these organs (10).

#### 2.1.5. ^99m^Tc-sestamibi (MIBI)

The role of ^99m^Tc-MIBI has been investigated as an imaging agent in patients with recurrent MTC. A study showed that ^99m^Tc-MIBI was more sensitive than CT for the detection of MTC recurrence in the neck and chest, but CT was more sensitive for lesions in the liver (27). Also, ^99m^Tc-diphosphonate bone scintigraphy had better sensitivity in the detection of bone metastases than ^99m^Tc-MIBI (27). Another study has demonstrated that the sensitivities of ^99m^Tc-MIBI, ^201^Tl and ^99m^Tc(V)-DMSA were 47%, 19% and 95%, respectively (28). 

#### 2.1.6. Radiolabelled Monoclonal Antibodies

Several monoclonal antibodies have been used to image patients with MTC. These include ^123^I-, ^131^I- and ^111^In-CEA (carcinoembryonic antigen) antibodies, both whole antibody and fragments, and ^111^In-anticalcitonin antibody (10). Results from imaging with monoclonal antibodies have been varied, ranging from 0% with anticalcitonin antibody to 78% with ^131^I-anti-CEA antibody (29). The results with labeled anti-CEA antibodies seem to be related to the aggressiveness of the neoplasm (29).

A study compared imaging with ^131^I-anti-CEA antibody and ^131^I-MIBG and showed significantly higher lesion detection with the antibody (30). Another study compared the results of imaging with ^131^I-MIBG, ^201^Tl and ^111^In-anti-CEA antibody and showed that imaging with the antibody yielded the best results, while another study showed that the sensitivity of imaging with ^99m^Tc(V)-DMSA and ^131^I-anti-CEA antibody was far superior to imaging with MIBG (14).

#### 2.1.7. Cholecystokinin (CCK)-B/Gastrin Receptor Imaging

Cholecystokinin (CCK) is a peptide hormone, originally discovered in the gastrointestinal tract in 1928 (31). Three types of CCK receptors have been identified so far: CCK1, CCK2, and CCK2i4sv receptors (31). A surprising high incidence of CCK2 receptors, reaching 90%, has been identified in MTC; whereas, differentiated thyroid cancers do not express CCK2 receptors (32). 

The first published clinical study with ^111^In-DTPA-minigastrin (MG0) was reported in 1999 (33). Since then several studies in patients with MTC have been performed with both gastrin-like and CCK-like peptides (^111^In-DTPA-MG0,^ 111^In-DOTA-MG11, ^111^In-DOTA-CCK8, ^99m^Tc-Demogastrin2) in the past few years (34, 35). The reported detection rate of ^111^In-DTPA-MG0 imaging in patients with MTC is up to 87% (35). Future studies are warranted to establish the optimal CCK2 receptor targeting peptide. 

### 2.2. Positron-Emitting Radiopharmaceuticals for PET Imaging

Positron Emission Tomography (PET) complements anatomic imaging by adding unique metabolic information to the characterization of malignancy. The method of ΡET/CT has the great advantage of combining functional and anatomic imaging at the same time, following image fusion.

It uses the ability of radiolabelled tracers to be taken-up by certain tumours, and thus selectively assesses the function of different metabolic pathways of the specific tissue. Positron-emitting isotopes frequently used for PET imaging include oxygen-15 (^15^O), nitrogen-13 (^13^N), carbon-11 (^11^C), and fluorine-18 (^18^F). 

The positron-emitting radiopharmaceuticals currently available for neuroendocrine tumors imaging may be divided into two groups: tracers which mark cell metabolism – [^18^F]FDG (fluorodeoxyglucose), [^18^F]DOPA (fluoro-dihydroxy-fluorophenylalanine), [^11^C]HTP ([^11^C]5-hydroxytryptophan), and tracers being specific ligands for receptors expressed on these cells- [^68^Ga]DOTA-peptides like [^68^Ga]DOTA-TOC and [^68^Ga]DOTA-TATE (36).

#### 2.2.1. ^18^ F-FDG

^18^F-fluorodeoxyglucose (^18^F-FDG) was the first tracer used, reflecting the increased glucose uptake in malignant tumours (36). During the past few years, numerous studies have demonstrated that the uptake of ^18^F-FDG is related to tumor grade and proliferation status in a wide variety of tumors (36). In general, low-grade, slowly proliferating tumors take up less ^18^F-FDG than poorly differentiated, rapidly growing tumors (36). Although 18F-FDG is certainly not the tracer of choice to study well differentiated neuroendocrine tumors, it has shown a higher sensitivity in patients with MTC when compared to single photon emission tracers (36-38). 

Several studies have now been undertaken using ^18^F-FDG in patients with elevated calcitonin level indicating recurrent MTC (37-42). It seems that ^18^F-FDG PET can play a major role in the follow-up of patients with postoperative elevated plasma calcitonin with detection of tumor remnant or recurrence (7, 38). 

The sensitivity of ^18^F- PET for recurrence and residual disease detection per patient is reported to be 44.1%-85%, and also provides additional information in a significant fraction of cases (up to 54%) (37, 39-42). However, ^18^F- PET is limited by poorer accuracy in the detection of small lesions, especially in the lung and liver (40). Comparing ^18^F- PET with conventional morphologic imaging methods (U/S, CT, MRI) and functional imaging methods with single-photon emitters in several studies, it can be noted that the ^18^F- PET revealed metastatic lesions in a higher percentage of patients (37, 38, 43). Other studies have suggested that ^18^F- PET imaging is more sensitive in patients with rapidly progressive disease than those with slowly rising calcitonin levels (39). 

In a study, the authors compared ^99m^Tc(V)-DMSA scintigraphy, ^111^In-DTPA-octreotide, ultrasound, CT, MRI, and ^18^F-FDG PET and concluded that ^18^F-FDG PET had the highest sensitivity in localizing metastatic disease (43). Another study showed that ^18^F-FDG PET was superior with better sensitivity than CT, MRI, and ^131^I-MIBG in localizing lymph node involvement in patients with known MTC and postoperatively elevated calcitonin levels (7). The location of disease recurrence greatly influences the selection of the appropriate imaging modality. ^18^F-FDG PET, in comparison with CT and MRI, demonstrates higher sensitivity for lesions in neck, supraclavicular, and mediastinal lymph nodes (7). However, CT shows higher efficacy for detection of liver and lung metastases; whereas, ^18^F-FDG PET and MRI were similar (7). 

A study performed on the prognostic value of ^18^F-FDG PET, showed that 55% of PET-positive patients succumbed to their disease; whereas, 93% of PET-negative patients remained disease free, with a follow-up period of about 44 months (44). Several researchers have correlated the detection rate of MTC recurrence with calcitonin levels and its doubling times (45). It is suggested that ^18^F-FDG PET imaging is more sensitive in patients with rapidly progressive disease than those with slowly rising calcitonin levels (45). Data from studies indicate that ^18^F-FDG PET or ^18^F-FDG ΡET/CT has its greatest utility in patients with calcitonin levels greater than 1000pg/ml (37, 41, 42, 45). Using an arbitrary cut-off of 1000pg/ml, the sensitivity for lesion detection in suspected residual, recurrent, or metastatic MTC increased, in three different studies from 62% to 78%, from 47.4% to 80%, and from 44.1% to 86.7%, respectively (14, 41, 42). These data also suggest that ^18^F-FDG PET and ^18^F-FDG PET/CT have limited usage in patients with low calcitonin levels (< 1000pg/mL), as the overall sensitivity was only 20%-36.8% (37, 41, 42, 45). It seems that the relatively low sensitivity in lesion detection in cases with low calcitonin levels is likely a reflection of microscopic disease or a smaller tumor burden. In general, it is known that limitations of ^18^F-FDG PET imaging in neuroendocrine tumors are the small size and the slow growth rate of the lesions (29). Studies have also shown that among the patients with MEN IIA syndrome the sensitivity of ^18^F-FDG PET/CT for MTC recurrence was significant lower (0-23%) and for patients with calcitonin levels < 2000pg/mL this fell to zero (0%) (41, 42). These findings are in accordance with the results of other studies which support that MEN IIA disease induces more indolent MTCs, and as ^18^F-FDG uptake relies on the biological aggressiveness of the tumor, the detection sensitivity of the method is low (41, 42, 46).

#### 2.2.2. Other Positron-Emitting Radiopharmaceuticals for PET Imaging

Given the heterogeneity of MTC phenotypic expression and the results of the studies analyzed above, it seems that [^18^F]FDG may not be the ideal radiotracer for imaging MTC. It is probably a suitable PET radiopharmaceutical to detect a small subset of biologically aggressive tumors that overexpress glucose transporter proteins. Other novel non-^18^F-FDG positron-emitting radiopharmaceuticals such as: a) ^18^F-fluorodihydroxyphenylalanine (^18^F-DOPA) and b) ^68^Ga-labelled somatostatin analogues. 

## 3. ^18^F-DOPA

^18^F-DOPA is the precursor to the neurotransmitters dopamine, norepinephrine and epinephrine, collectively known as catecholamines. PET with ^18^F-DOPA, provides a functional approach of pathologies, organs or tissues where enhanced intracellular transport and decarboxylation of the amino acid dihydroxyphenylalanine is the diagnostic target, allowing the visualization of the sympathetic cells (47, 48).

In most of the comparative studies the sensitivity of ^18^F-DOPA PET is higher than ^18^F-FDG PET in detection of MTC recurrence (47, 48). In 2001, investigators have reported that the sensitivity of ^18^F-DOPA PET was 63% for MTC detection, while more recent studies have shown higher rates of sensitivity between 74% and 87% (47-50). Some authors recommend ^18^F-DOPA PET as a one-stop diagnostic procedure to provide both functional and morphological data to select those patients who may benefit from reoperation with curative intent (50, 51). The difference in ^18^F-DOPA uptake seems to be related to MTC differentiation and proliferation (52).

Other data have supported that the combination of ^18^F-DOPA and ^18^F-FDG PET may give the highest sensitivity and specificity (50). The veritable cut-off value of calcitonin levels that could be predictive of a positive ^18^F-DOPA PET scan still remains controversial. Some authors have suggested a value >500 ng/l, although others suggested a lower value> 150 pg/ml (49, 51). Some investigators suggested the use of ^18^F-DOPA PET/CT as the first-choice examination in biochemically recurrent MTC because it seems that may offer increased sensitivity for identifying recurrence, assessing prognosis and guiding selection on appropriate treatment (52). 

The radiolabelled metabolite ^18^F-fluorodopamine (^18^F-DA) may be an alternative radiopharmaceutical to ^18^F-DOPA PET, as it has been shown to localize an MTC metastasis in a patient with MEN IIA (53). ^18^F-DA has been shown to be sensitive for the evaluation of NETs of chromaffin origin, pheochromocytomas and paragangliomas but larger series are required for MTC detection (54, 55)

## 4. ^68^ Ga-labelled Somatostatin Analogues

Somatostatin analogues labelled with positron-emitting radionuclides are used for imaging with PET cameras or hybrid PET/CT cameras with great potential due to two advantages they have over γ-emitting analogues (8). The most used radioisotope is ^68^Gallium, which is available from an in-house generator, rendering its production independent of an onsite cyclotron (6). ^68^Ga labelled somatostatin analogues (^68^Ga-DOTA-TOC or ^68^Ga-DOTA-NOC) represent also a promising tool for evaluation of the expression of somatostatin receptors in patients with metastatic neuroendocrine tumors in which therapy with ^177^Lu- or ^90^Y-labelled DOTA-TATE is planned (56, 57). 

A study has shown that ^68^Ga-DOTATATE PET/CT had a sensitivity of 72% in detection of MTC recurrence in patients with elevated calcitonin levels postoperatively, and this rate was similar to ^18^F-FDG PET/CT sensitivity of 78% (58). The researchers concluded that ^68^Ga-DOTATATE PET/CT could be a useful complementary imaging method which could identify patients suitable for targeted radionuclide somatostatin analogue therapy (58). A very recent study compared ^18^F-FDG, ^18^F-DOPA and ^68^Ga-somatostatin analogues PET/CT imaging in patients with residual/recurrent MTC suspected on the basis of elevated serum calcitonin levels and showed that ^18^F-DOPA PET/CT was the most useful imaging method for detecting recurrent MTC lesions performing better than ^18^F-FDG and ^68^Ga-somatostatin analogue PET/CT (59). In another retrospective study that detected the extent of disease in MTC using ^68^Ga-DOTATATE and ^18^F-FDG PET/CT there was nonsignificant difference in per-patient sensitivities, 72.2% and 77.8%, respectively (58).

## 5. Conclusions

There is no single sensitive diagnostic imaging method to reveal all MTC recurrences. Conventional morphologic imaging methods (US, CT, MRI) frequently fail to reveal the recurrent lesions. Some new radiopharmaceuticals are under investigation and others can be considered either of historical or experimental value and the use of others, like ^99m^Tc(V)-DMSA, is going to be abandoned. The sensitivity of ^123^Ι/^131^Ι-MIBG and Octreoscan, the most used radiopharmaceuticals in neuroendocrine tumors, is low and has been reported to be between 30% and 71%. It seems that ^18^F-FDG PET or PET/CT can play a major role in the follow-up of patients with postoperative elevated plasma calcitonin and the sensitivity for recurrence and residual disease detection per patient is reported to be 44.1-85%. ^18^F-FDG PET also provides additional information in a significant fraction of cases (up to 54%). Preliminary data suggest that the use of other PET tracers, such as ^18^F-DOPA and ^68^Ga-DOTATOC or ^68^Ga-DOTATATE, may provide a better lesion detection rate than does ^18^F-FDG.
